# Plant-Derived miR-55 Alleviates Liver Fibrosis by Disrupting the CK2α/SMO Complex and Promoting SMO Ubiquitination

**DOI:** 10.3390/ijms27020748

**Published:** 2026-01-12

**Authors:** Lei Wu, Jing Yang, Anqi Li, Yuqiang Zhao, Qing Liu, Zhenbo Li, Yihan Liu, Peng Tang, Rui Wang

**Affiliations:** 1 College of Pharmacy, Heilongjiang University of Chinese Medicine, Harbin 150040, China; wl2048972176@163.com (L.W.);; 2 College of Basic Medical Science, Heilongjiang University of Chinese Medicine, Harbin 150040, China; yangjingdx@sina.com (J.Y.);; 3 College of Biomedicine and Health, Huazhong Agricultural University, Wuhan 430070, China; 4 College of Life Science and Technology, Huazhong Agricultural University, Wuhan 430070, China; 5 Science and Technology Innovation Center, Guangzhou University of Chinese Medicine, Guangzhou 510006, China; 6 Key Laboratory of Basic and Application Research of Beiyao, Heilongjiang University of Chinese Medicine, Ministry of Education, Harbin 150040, China

**Keywords:** liver fibrosis, plant miR-55, cross-species regulation, CK2α/SMO, ubiquitination, targeted therapy

## Abstract

The development of RNA-based drugs for MAFLD-related fibrosis is severely hampered by the poor oral bioavailability of nucleic acids. This study employed a novel, patent-protected LNP formulation to orally deliver plant-derived miR-55 and investigate its therapeutic potential, focusing on its novel mechanism of action via the CK2α/SMO interaction. In a rat model established with a methionine-choline-deficient diet, orally administered miR-55 markedly improved liver injury, lipid dysregulation, oxidative stress, and pathological collagen deposition. The anti-fibrotic efficacy was quantitatively confirmed by a significant reduction in hepatic hydroxyproline content and downregulation of key fibrogenic genes (*Col1a1*, *Col3a1*, *TIMP-1*, *TGF-β1*, *CTGF*) and pro-inflammatory cytokines (TNF-α, IL-6), achieving effects comparable to the full Ge Xia Zhu Yu Decoction. Mechanistically, both bioinformatic prediction and in vivo validation indicated that miR-55 is predicted to target CK2α. This targeting suppressed CK2α expression and disrupted the endogenous CK2α-SMO complex, thereby promoting the ubiquitin-mediated degradation of SMO—a previously unreported mechanism. This cascade inhibited the downstream Gli1 pathway and downregulated pro-fibrotic and pro-angiogenic factors (VEGF, PDGF), thereby providing a comprehensive mechanistic basis for the therapeutic effects. This study is the first to provide evidence that orally delivered, plant-derived miR-55 may act as a natural modulator that potentially through disrupting the CK2α/SMO interaction via a unique complex disruption-promoted degradation mechanism, attenuating Hedgehog signaling and alleviating liver fibrosis. These findings offer important insights into cross-kingdom regulation and highlight miR-55 as a potential targeted therapeutic candidate.

## 1. Introduction

Metabolic Dysfunction-Associated Fatty Liver Disease (MAFLD) is now the most prevalent chronic liver disorder worldwide, and its progression and prognosis depend critically on the degree of liver fibrosis [1,2]. Despite its significant clinical burden, no pharmacological therapy is currently approved to effectively reverse liver fibrosis, underscoring an urgent need for novel and targeted therapeutic strategies [3].

Hepatic Stellate Cell (HSC) activation is a central event in hepatic fibrogenesis. Upon chronic injury, HSCs undergo activation, a process characterized by the induction of α-SMA and their trans differentiation into proliferative, contractile myofibroblasts [4]. This activation is primarily driven by key cytokines, most notably *TGF-β1*, which acts as the most potent profibrogenic signal [5]. Crucially, the fibrogenic actions of *TGF-β1* are largely mediated through its key downstream effector, *CTGF*, which acts as a central amplifier and executor, directly stimulating the sustained production of Extracellular Matrix (ECM) components. Activated HSCs are the main source of excessive ECM deposition, predominantly *Col1a1* and *Col3a1*, which disrupts the normal liver architecture [6]. Concurrently, the ECM degradation pathway is suppressed, largely through the overexpression of *TIMP-1*, creating a pathologic imbalance that favors matrix accumulation [7]. In this context, the Hedgehog (Hh) signaling pathway has been shown to be a key driver of HSC activation and liver fibrosis progression.

The fibrotic microenvironment is further exacerbated by hypoxia and pathological angiogenesis. HIF-1α, a master regulator of oxygen homeostasis, accumulates in the injured liver and transcriptionally activates a suite of pro-fibrotic genes, including VEGF [8]. VEGF, in turn, drives the formation of abnormal vasculature, while PDGF emerges as a potent mitogen for HSCs [9]. This HIF-1α/VEGF/PDGF axis forms a vicious cycle that perpetuates HSC activation and fibrosis progression.

Notably, CK2α not only participates in MAFLD pathogenesis but also acts as a crucial regulator of Hh signaling by directly binding to and stabilizing SMO, its core transducer [10,11]. Specifically, Fan et al. demonstrated that CK2α binding to SMO inhibits the ubiquitin-mediated degradation of SMO, thereby sustaining downstream pro-fibrotic signaling [12]. This discovery positions the CK2α/SMO protein–protein interaction as a promising and previously unexplored therapeutic target for anti-fibrotic intervention.

Furthermore, the fibrotic process is propelled by a chronic inflammatory microenvironment. Key pro-inflammatory cytokines, such as TNF-α and IL-6, are markedly upregulated in MAFLD, where they act as potent initiators and amplifiers of HSC activation and the subsequent fibrogenic response [13,14].

The development of RNA-based therapeutics represents a frontier in precision medicine, yet its application is often limited by the challenge of poor oral bioavailability, which hinders clinical translation. While plant-derived miRNAs possess inherent stability due to 3′-terminal 2′-O-methylation and have been reported to exert cross-kingdom regulatory effects, achieving efficient systemic delivery via oral administration remains a critical hurdle [15,16,17,18]. This paradigm was notably demonstrated by the seminal finding of plant miRNAs in mammalian sera and their potential to regulate host genes [19]. However, achieving efficient systemic delivery via oral administration remains a critical hurdle. To overcome this central challenge, we employed a proprietary oral delivery technology developed under two national authorized invention patents [20,21]. This LNP system protects the miRNA from enzymatic and acidic degradation in the gastrointestinal tract and enhances intestinal absorption, thereby ensuring systemic bioavailability. The Lipid Nanoparticle (LNP) platform has been clinically validated as a robust and safe delivery vehicle for nucleic acids, including siRNA therapeutics [22,23], underscoring its translational potential for oral RNAi therapy.

Our preliminary bioinformatics screening, leveraging this advanced delivery platform, identified a stably enriched miRNA from the traditional Chinese medicine formula Ge Xia Zhu Yu Decoction—plant-derived miR-55—as a potential candidate for improving metabolic and oxidative stress in MAFLD. Intriguingly, target prediction analysis suggested that miR-55 may target CK2α mRNA.

The selection of plant-derived miR-55 for this investigation, rather than a synthetic miRNA mimic, was guided by the following considerations. First, the specific computational prediction that miR-55 may target CK2α (Supplementary Figure S1) directly links it to the potential modulation of the CK2α/SMO complex—a novel and promising target in fibrosis [12]. This provides a clear, mechanism-based rationale distinct from using synthetic miRNAs to target general disease pathways. Second, the inherent 3′-terminal 2′-O-methylation of plant miRNAs confers enhanced biostability compared to unmodified synthetic RNAs [24,25], making miR-55 particularly compatible with and resilient for our oral LNP delivery strategy. Third, focusing on a miRNA enriched in Ge Xia Zhu Yu Decoction, a formula with documented anti-fibrotic efficacy [26,27,28], allows us to explore the molecular basis of a clinically relevant intervention, offering a unique translational bridge between traditional medicine and modern targeted therapy.

Based on this converging evidence, we hypothesized that plant-derived miR-55, delivered via our patented LNP system, could serve as a natural, orally available therapeutic agent whose mechanism may involve the suppression of CK2α. We hypothesized a mechanism whereby miR-55, upon its association with reduced CK2α expression, disrupts the protective CK2α-SMO complex. This disruption was anticipated to promote the ubiquitination and degradation of SMO, ultimately leading to the inhibition of the downstream Hh/Gli1 signaling axis and the consequent attenuation of the multifaceted pro-fibrotic response, encompassing HSC activation (α-SMA), ECM imbalance (*Col1a1*, *Col3a1*, *TIMP-1*), cytokine signaling (*TGF-β1*), and pathological angiogenesis (HIF-1α, VEGF, PDGF).

To test this hypothesis, we employed a rat model of MAFLD-related fibrosis and pursued two primary objectives: (1) to validate the in vivo anti-fibrotic efficacy of orally administered miR-55 delivered by our patented LNP system, and (2) to explore its potential mechanism of action, particularly its association with the disruption of the CK2α/SMO complex and subsequent promotion of SMO ubiquitination. This study investigates a plant miRNA that may target a critical protein–protein interaction interface, offering not only a potential mechanistic insight into cross-kingdom regulation but also a foundation for developing RNA-based precision therapies against liver fibrosis.

**Figure 1:**
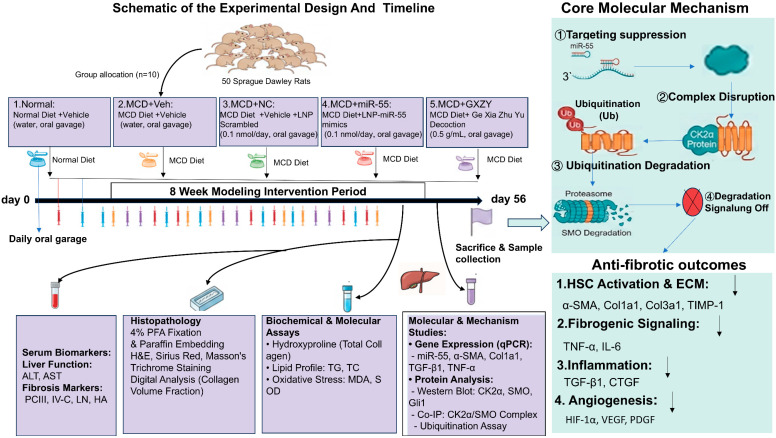
Schematic of the experimental design and proposed mechanism of plant-derived miR-55 against liver fibrosis. (**Left**) In vivo workflow: Rats were allocated into five groups and treated for 8 weeks alongside MCD diet-induced modeling. Interventions were administered daily via oral gavage, followed by comprehensive endpoint analyses. (**Right**) Molecular mechanism: Orally delivered, LNP-formulated miR-55 targets hepatic CK2α, disrupting the CK2α-SMO complex and promoting SMO ubiquitination and degradation, thereby inhibiting the pro-fibrotic Hedgehog signaling pathway.

The overall experimental design and the proposed molecular mechanism are summarized in Figure 1.

## 2. Results

### 2.1. miR-55 Exhibits High Stability in Simulated Gastrointestinal Environment

**Figure 2:**
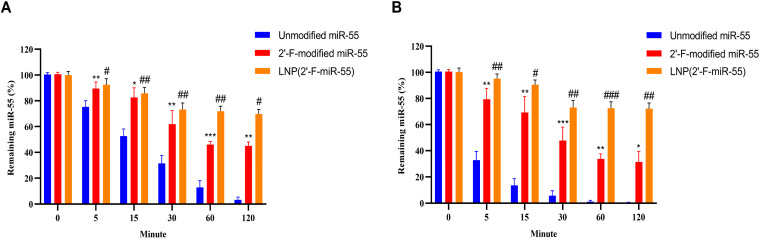
Stability of miR-55 in simulated gastrointestinal fluids. (**A**) Stability of LNP-formulated miR-55 in simulated gastric fluid (SGF, pH 2.0). (**B**) Stability of LNP-formulated miR-55 in simulated intestinal fluid (SIF, pH 6.8). Data are expressed as mean ± SD (*n* = 6). * *p* < 0.05, ** *p* < 0.01, *** *p* < 0.001 compared with the 0 min time point within the same group; # *p* < 0.05, ## *p* < 0.01, ### *p* < 0.001 compared with unmodified miR-55 (one-way ANOVA with Tukey’s post hoc test).

Prior to in vivo evaluation, we compared the stability of unmodified, 2′-F-modified, and LNP-encapsulated 2′-F-miR-55 under simulated gastrointestinal conditions. After 2 h incubation in simulated gastric fluid, only 3.2% of unmodified miR-55 remained intact, while 2′-F-miR-55 and LNP-encapsulated 2′-F-miR-55 retained 44.9% and 69.7% integrity, respectively, both significantly higher than the unmodified group, with the LNP-formulated group also being significantly more stable than the 2′-F-miR-55 alone. Similarly, in simulated intestinal fluid, unmodified miR-55 was nearly completely degraded (1.8% remaining), whereas 2′-F-miR-55 and LNP-formulated groups maintained 31.4% and 72.3% integrity, respectively, both significantly higher than the unmodified control, and the LNP group again showed significantly greater protection than the 2′-F-miR-55 group (Figure 2A,B).

### 2.2. miR-55 Attenuates MCD Diet-Induced Weight Loss in MAFLD Rats

**Figure 3:**
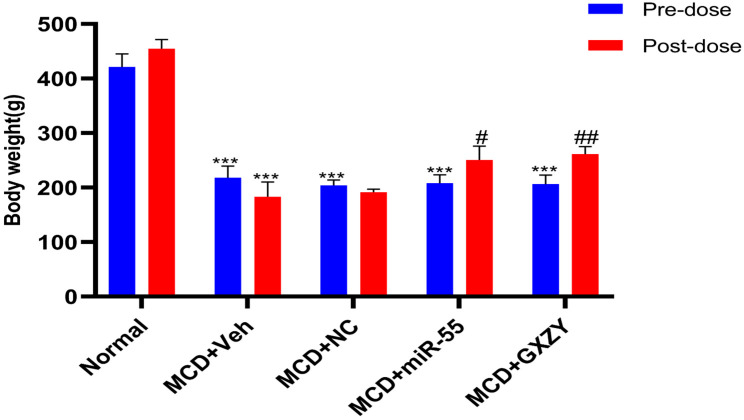
Body weight changes in experimental rats. Normal diet (Normal); MCD diet + vehicle (MCD + Veh); MCD diet + negative control miRNA (MCD + NC); MCD diet + miR-55 mimics (MCD + miR-55); MCD diet + Ge Xia Zhu Yu Decoction (MCD + GXZY). Data are expressed as mean ± SD (*n* = 10). *** *p* < 0.001 compared with the Normal group; # *p* < 0.05, ## *p* < 0.01 compared with the MCD + Veh group (one-way ANOVA with Tukey’s post hoc test).

As shown in Figure 3, rats fed an MCD diet exhibited a significant reduction in body weight compared to the Normal control (*p* < 0.001), confirming successful induction of the MAFLD model. Treatment with a nonspecific negative control miRNA (MCD + NC group) did not ameliorate weight loss and was not significantly different from the model group (MCD + Veh). In contrast, administration of miR-55 led to a significant restoration of body weight relative to the MCD + Veh group (*p* < 0.05). This therapeutic effect was comparable to that achieved by the positive control, MCD + GXZY, which also significantly improved body weight compared to the MCD + Veh group (*p* < 0.01). These results indicate that synthetic miR-55 mimics effectively reproduce the beneficial effect of the full Ge Xia Zhu Yu Decoction formulation on improving the systemic condition in model animals.

### 2.3. miR-55 Ameliorates Liver Function, Lipid Metabolism, and Oxidative Stress in MAFLD Rats

**Figure 4:**
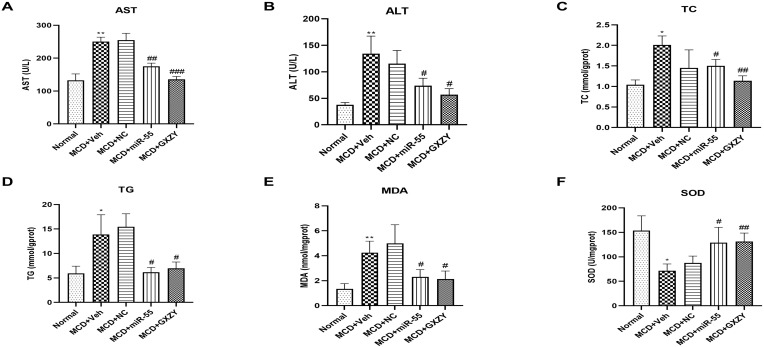
Effects of miR-55 intervention on liver injury, lipid metabolism, and oxidative stress. (**A**,**B**) The activities of serum AST and ALT. (**C**) Hepatic TC, (**D**) hepatic TG, (**E**) hepatic MDA, and (**F**) hepatic SOD. Data are presented as mean ± SD (*n* = 10). Statistical comparisons were performed using one-way ANOVA followed by Tukey’s post hoc test. # *p* < 0.05, ## *p* < 0.01, ### *p* < 0.001 compared with the Normal group; * *p* < 0.05, ** *p* < 0.01 compared with the MCD + Veh group. Treatment groups: Normal diet (Normal); MCD diet + vehicle (MCD + Veh); MCD diet + negative control miRNA (MCD + NC); MCD diet + miR-55 mimics (MCD + miR-55); MCD diet + Ge Xia Zhu Yu Decoction (MCD + GXZY).

Serological analysis indicated successful induction of liver injury by the MCD diet, as evidenced by significantly elevated serum ALT and AST activities in the model group (MCD + Veh) relative to the normal control (Normal) (*p* < 0.01). Treatment with miR-55 significantly reduced ALT (*p* < 0.05) and AST levels (*p* < 0.01), demonstrating hepatoprotective effects comparable to those of the positive control Ge Xia Zhu Yu Decoction (MCD + GXZY) (Figure 4A,B).

Analysis of hepatic tissue lipids revealed that TG and TC content were markedly increased in the MCD + Veh group compared to the Normal group (*p* < 0.05). Importantly, miR-55 administration effectively attenuated this lipid accumulation in the liver (*p* < 0.05), indicating improved hepatic lipid metabolism (Figure 4C,D).

Furthermore, miR-55 alleviated hepatic oxidative stress, as reflected by significantly reduced MDA levels and increased SOD activity in liver tissues (*p* < 0.05; Figure 4E,F).

### 2.4. miR-55 Alleviates Liver Fibrosis: Evidence from Histopathology, Biochemical and Molecular Markers

#### 2.4.1. Histopathological Improvement and Collagen Deposition Quantification

**Figure 5:**
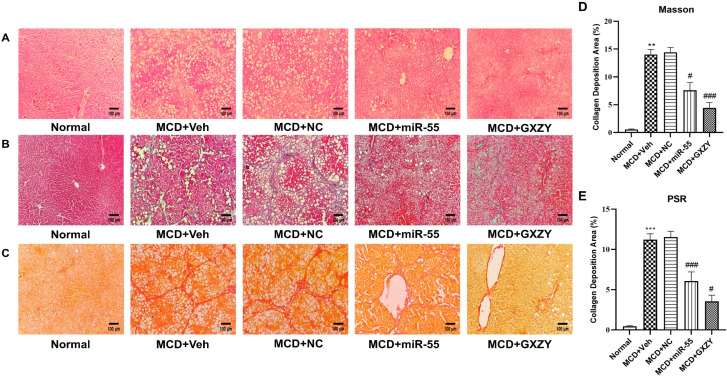
Histopathological improvement and collagen deposition quantification. (**A**) Representative H&E staining images. (**B**) Representative Masson’s trichrome staining images. (**C**) Representative Sirius red staining images. (**D**,**E**) Quantitative analysis of CVF from Sirius red and Masson’s trichrome staining, respectively. Data are presented as mean ± SD. Statistical comparisons were performed using one-way ANOVA followed by Tukey’s post hoc test. # *p* < 0.05, ### *p* < 0.001 compared with Normal group; ** *p* < 0.01, *** *p* < 0.001 compared with the MCD + Veh group.

Histological evaluation by H&E staining (Figure 5A) showed intact lobular architecture in the Normal group. The MCD + Veh group displayed severe steatosis, inflammatory cell infiltration, and fibrous connective tissue hyperplasia. Both the MCD + miR-55 and MCD + GXZY groups showed notable histological improvement.

Masson’s trichrome (Figure 5B) and Sirius red (Figure 5C) staining revealed prominent collagen deposition in the MCD + Veh and MCD + NC groups, which was markedly reduced by miR-55 treatment. To provide rigorous quantification, we performed digital image analysis of the Sirius red and Masson’s trichrome stains. The results demonstrated that miR-55 treatment significantly reduced the CVF compared to the MCD + Veh group (*p* < 0.01; Figure 5D,E).

#### 2.4.2. miR-55 Reduces Circulating Levels of Established Fibrosis Markers

**Figure 6:**
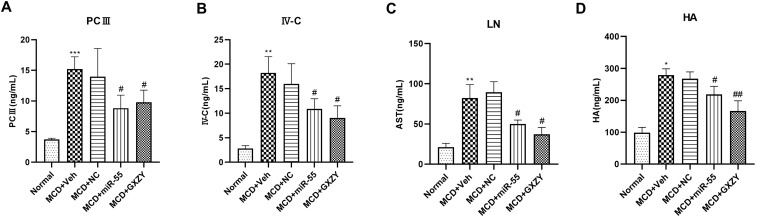
miR-55 reduces serum levels of established fibrosis markers. (**A**–**D**) Serum levels of PCIII, IV-C, LN, and HA. Data are presented as mean ± SD (*n* = 10). Statistical comparisons were performed using one-way ANOVA followed by Tukey’s post hoc test. * *p* < 0.05, ** *p* < 0.01, *** *p* < 0.001 compared with the Normal group. # *p* < 0.05, ## *p* < 0.01 compared with the MCD + Veh group.

To further evaluate the anti-fibrotic effect of miR-55 at the systemic level, we measured serum levels of established clinical fibrosis markers, including PCIII, IV-C, LN, and HA. The MCD + Veh group exhibited significantly elevated levels of all four markers compared to the Normal group (*p* < 0.001). Treatment with miR-55 significantly reduced the serum levels of PCIII, IV-C, LN, and HA (*p* < 0.01 for PCIII and IV-C; *p* < 0.05 for LN and HA) compared to the MCD + Veh group, demonstrating its potent anti-fibrotic activity (Figure 6A–D).

#### 2.4.3. Reduction in Hepatic Collagen Content and Profibrogenic Gene Expression

**Figure 7:**
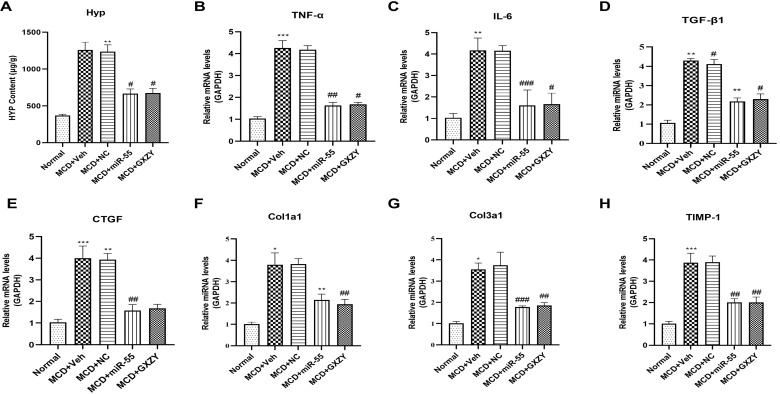
miR-55 suppresses hepatic pro-inflammatory and profibrogenic gene expression. (**A**) Hepatic hydroxyproline content. (**B**,**C**) mRNA expression levels of pro-inflammatory cytokines TNF-α and IL-6. (**D**–**H**) mRNA expression levels of profibrogenic mediators *TGF-β1*, *CTGF*, *Col1a1*, *Col3a1*, and *Timp-1*. Data are presented as mean ± SD (*n* = 6). Statistical comparisons were performed using one-way ANOVA followed by Tukey’s post hoc test. # *p* < 0.05, ## *p* < 0.01, ### *p* < 0.001 compared with Normal group; * *p* < 0.05, ** *p* < 0.01, *** *p* < 0.001 compared with MCD + Veh group.

miR-55 treatment significantly reduced hepatic hydroxyproline content compared to the MCD + Veh group (*p* < 0.01; Figure 7A). Q-PCR analysis revealed significant upregulation of TNF-α and IL-6 mRNA expression in the MCD + Veh group relative to the Normal group (*p* < 0.001). miR-55 treatment significantly reduced mRNA levels of both TNF-α and IL-6 (*p* < 0.01; Figure 7B,C).

Analysis of key mediators within the core fibrogenic cascade showed that miR-55 administration significantly suppressed mRNA expression of the fibrogenic cytokine *TGF-β1* (*p* < 0.01; Figure 7D) and its downstream executor *CTGF* (*p* < 0.01; Figure 7E). Expression of the primary ECM components *Col1a1* (*p* < 0.001; Figure 7F) and *Col3a1* (*p* < 0.01; Figure 7G), along with the protease inhibitor *Timp-1* (*p* < 0.01; Figure 7H), were also significantly downregulated by miR-55 treatment.

### 2.5. Mechanisms miR-55 Targets the CK2α/SMO Axis: From Prediction to Mechanism

#### 2.5.1. miR-55 Is Associated with Suppression of CK2α Expression: From Prediction to In Vivo Observation

**Figure 8:**
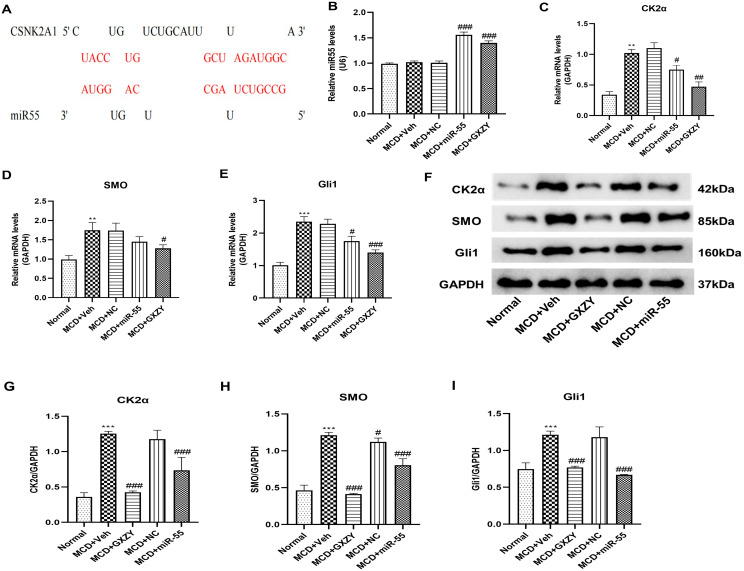
miR-55 targets CK2α and suppresses the CK2α/SMO/Gli1 axis in vivo. (**A**) Schematic of the predicted binding site between miR-55 and the 3′-UTR of human CK2α mRNA. The red highlight indicates the predicted miR-55 binding seed region. (**B**) Hepatic relative expression level of synthetic miR-55. (**C**–**E**) mRNA expression levels of CK2α, SMO, and Gli1. (**F**) Representative Western blot images. (**G**–**I**) Densitometric quantification of protein expression of CK2α (**G**), SMO (**H**), and Gli1 (**I**) from (**F**). Normal, control group; MCD + Veh, model group; MCD + NC, negative control group; MCD + miR-55, intervention group; MCD + GXZY, positive control group. Data are presented as mean ± SD (*n* = 6). Statistical comparisons were performed using one-way ANOVA followed by Tukey’s post hoc test. # *p* < 0.05, ## *p* < 0.01, ### *p* < 0.001 compared with Normal group; ** *p* < 0.01, *** *p* < 0.001 compared with the MCD + Veh group.

To investigate the mechanism underlying the therapeutic effects of miR-55, we performed a bioinformatic analysis using the established psRobot and RNAhybrid algorithms [29,30]. As detailed in Supplementary Figure S1, this analysis predicted with high confidence that plant-derived miR-55 possesses a thermodynamically stable binding site within the 3′-UTR of human CK2α mRNA, with a minimum free energy of −30.4 kcal/mol and a supporting psRobot score of 1.5. This in silico evidence suggested a potential regulatory relationship, a concept that is schematically summarized in Figure 8A. We subsequently explored this prediction in vivo.

qPCR analysis confirmed a marked increase in the levels of synthetic miR-55 in the livers of MCD + miR-55-treated rats compared to the MCD + Veh group (*p* < 0.001; Figure 8B), confirming successful in vivo delivery. Furthermore, administration of MCD + miR-55 significantly reduced the mRNA expression of both CK2α and its downstream transcription factor Gli1 (*p* < 0.05; Figure 8C,E), while SMO mRNA levels remained statistically unchanged (Figure 8D). This distinct expression profile is consistent with the bioinformatic prediction that miR-55 may regulate CK2α, thereby potentially influencing SMO indirectly.

Western blot analysis further supported these observations at the protein level (Figure 8F). MCD + miR-55 treatment led to significant reductions in the protein expression of CK2α (*p* < 0.001), SMO (*p* < 0.05), and Gli1 (*p* < 0.01) relative to the MCD + Veh group (Figure 8G–I). Collectively, these results Provide evidence that MCD + miR-55 treatment is associated with effective suppression of the activated CK2α/SMO/Gli1 signaling axis.

#### 2.5.2. miR-55 Disrupts the CK2α–SMO Complex and Promotes Ubiquitin-Mediated Degradation of SMO

**Figure 9:**
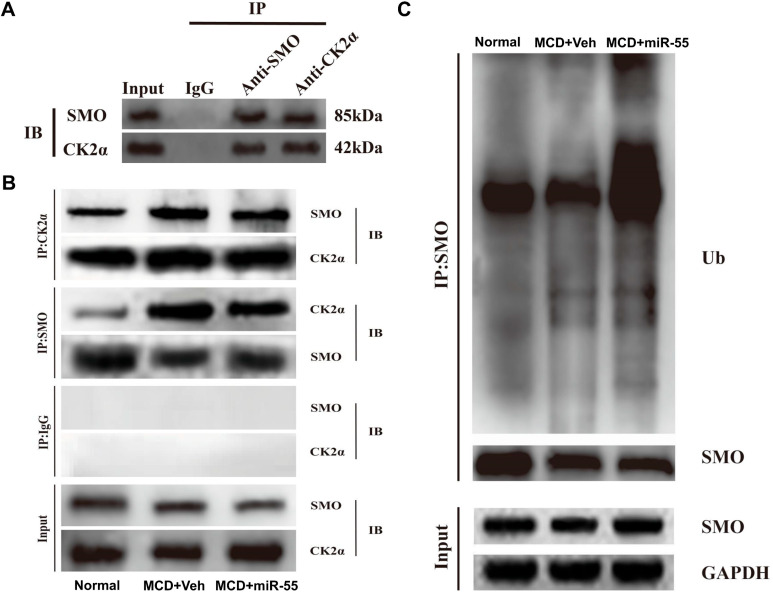
miR-55 disrupts the CK2α-SMO complex and promotes SMO ubiquitination. (**A**) Co-IP confirms the endogenous interaction between CK2α and SMO. (**B**) miR-55 treatment disrupts the CK2α-SMO interaction. (**C**) miR-55 promotes polyubiquitination of SMO. IP, immunoprecipitation; IB, immunoblot; Ub, ubiquitin. Data are representative of three independent experiments.

To investigate whether miR-55 downregulates CK2α expression and consequently destabilizes the CK2α–SMO interaction, Co-IP assays were performed. The results confirmed endogenous binding between CK2α and SMO in fibrotic liver tissues (Figure 9A). miR-55 treatment markedly attenuated this protein interaction (Figure 9B).

Further analysis of the mechanism of SMO degradation was conducted through ubiquitination assays following SMO immunoprecipitation. The assays indicated a significant increase in polyubiquitinated SMO upon miR-55 treatment (Figure 9C). These results provide evidence that miR-55 disrupts the CK2α–SMO complex and promotes ubiquitin-mediated proteasomal degradation of SMO.

#### 2.5.3. miR-55 Suppresses Pro-Fibrotic and Pro-Angiogenic Downstream Responses

**Figure 10:**
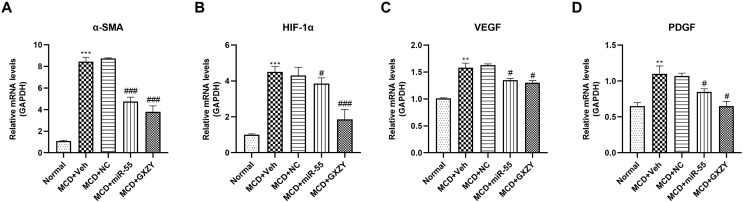
miR-55 inhibits the expression of pro-fibrotic and pro-angiogenic factors. miRNA expression levels of (**A**) α-SMA, (**B**) HIF-1α, (**C**) VEGF, and (**D**) PDGF in liver tissues. Data are presented as mean ± SD (*n* = 6). Statistical comparisons were performed using one-way ANOVA followed by Tukey’s post hoc test. # *p* < 0.05, ### *p* < 0.001 compared with Normal group; ** *p* < 0.01, *** *p* < 0.001 compared with the MCD + Veh group.

To evaluate the functional consequences of the observed molecular events, we analyzed the expression of key downstream effector genes. qPCR analysis revealed that miR-55 treatment significantly reduced mRNA levels of the hepatic stellate cell activation marker α-SMA, the hypoxia-inducible factor HIF-1α, and the angiogenic factors VEGF and PDGF compared to the MG group (*p* < 0.05; Figure 10). These results indicate that miR-55, through targeting the CK2α–SMO–Gli1 axis, effectively suppresses transcriptional programs driving fibrogenesis and pathological angiogenesis.

## 3. Discussion

Liver fibrosis is a central driver in the progression of MAFLD, characterized by persistent activation of HSCs and excessive deposition of ECM [31,32,33]. Although current anti-fibrotic drug development favors multi-target strategies, the identification of specific molecular interventions capable of precisely modulating key upstream nodes remains a major challenge [34,35]. This study provides novel evidence that plant-derived miR-55—originating from the traditional Chinese formula Ge Xia Zhu Yu Decoction—can be orally delivered and modulates the CK2α/SMO/Gli1 signaling axis, significantly ameliorating MAFLD-associated liver fibrosis. The anti-fibrotic efficacy was clearly demonstrated through a multi-level quantitative assessment. Beyond the evident improvement in histopathology, digital morphometric analysis confirmed a significant reduction in collagen deposition, which was further validated by a marked decrease in hepatic hydroxyproline content, a biochemical gold standard for total collagen [36].

At the molecular level, this resolution of fibrosis was driven by the coordinated downregulation of key fibrogenic mediators. Beyond the suppression of the primary ECM components *Col1a1* and *Col3a1* and the critical protease inhibitor *TIMP-1*, our study provides crucial evidence that miR-55 significantly inhibits the *TGF-β1*/*CTGF* axis. *CTGF* is widely recognized as a central downstream effector of *TGF-β1*, responsible for amplifying and perpetuating its pro-fibrotic signal, leading to sustained collagen deposition [37]. The marked reduction in *CTGF* expression upon miR-55 treatment indicates that the therapeutic intervention effectively disrupts this key fibrogenic amplification loop.

Furthermore, our study elucidates the beneficial role of miR-55 in mitigating hepatic oxidative stress, which constitutes a key pathogenic component in MAFLD progression [38]. As shown in Figure 4F, miR-55 intervention significantly restored the activity of SOD in the liver of MCD diet-fed rats. The assay measured total SOD activity in liver homogenates, which reflects overall antioxidant capacity. The diminished SOD activity in the model group is consistent with the established notion of oxidative/antioxidant imbalance in MAFLD, likely resulting from sustained metabolic stress, mitochondrial dysfunction, and exhaustion of the antioxidant defense system [39]. The recovery of SOD activity by miR-55 suggests an enhancement of the hepatic antioxidant machinery. This protective effect may be achieved through multiple mechanisms: first, by suppressing the CK2α/SMO/Gli1 axis and its downstream effectors (HIF-1α, TNF-α, IL-6), miR-55 likely reduces the overall burden of ROS generation [40]. Second, the improvement in hepatocyte function and mitochondrial integrity, along with the attenuation of lipid accumulation and lipotoxicity, may contribute to decreased production of lipid peroxidation products, thereby alleviating the consumption of the SOD system [41]. Together, these actions represent a cell-protective mechanism under conditions of lipid peroxidation activation and accumulation, further disrupting the vicious cycle wherein oxidative stress drives HSC activation and fibrogenesis [40]. Thus, beyond its association with the CK2α/SMO interaction, miR-55 exhibits a pleiotropic regulatory advantage by improving redox homeostasis, providing a more comprehensive mechanistic basis for its therapeutic potential against MAFLD-related fibrosis [42].

Critically, the success of this oral RNAi therapy was contingent upon overcoming the fundamental challenge of systemic delivery. The LNP formulation employed herein provided robust gastrointestinal protection (Figure 2) and facilitated efficient hepatic accumulation of miR-55 (Figure 8B), leveraging the natural liver tropism of LNPs [23,43,44].

In contrast to endogenously dysregulated miRNAs in MAFLD, such as miR-122 and miR-21 [29,45,46], plant miRNAs exhibit enhanced stability due to 3′ terminal 2′-O-methylation modifications, enabling them to resist digestive degradation, enter systemic circulation, and exert biological functions in mammals [30,47]. Our patented delivery strategy further enhances the oral bioavailability of miR-55. While plant miRNAs are commonly thought to function through target mRNA silencing akin to endogenous miRNAs [48], this study suggests a potential mechanism that may extend beyond simple suppression of CK2α expression: convergent evidence from bioinformatic prediction and in vivo validation indicates that miR-55 treatment leads to disruption of the functional CK2α–SMO protein complex, thereby promoting ubiquitination and degradation of SMO. This suggests the possibility of a mechanism that extends beyond conventional gene silencing towards influencing critical protein–protein interaction, potentially expanding the understanding of cross-kingdom regulatory mechanisms that may be employed by plant miRNAs.

CK2α activates Hh signaling and promotes HSC activation by directly binding to and stabilizing SMO. While previous studies, including the work by Fan et al. [12], have demonstrated that broad-spectrum pharmacological inhibition of CK2α can alleviate fibrosis, such approaches inherently lack specificity and carry risks of off-target effects. In contrast to these conventional kinase-targeting inhibitors, our study suggests that miR-55 may function as a modulator whose therapeutic effect is mediated, at least in part, through the disruption of the CK2α–SMO protein–protein interaction interface. This strategy of disrupting a specific PPI, rather than globally inhibiting kinase activity, could offer a potential for enhanced selectivity and a lower risk of interfering with the numerous physiological functions of CK2α. The data presented here confirm that miR-55 significantly reduces α-SMA expression and collagen deposition—effects consistent with the documented anti-fibrotic activity of Ge Xia Zhu Yu Decoction [49,50]. Crucially, we provide molecular evidence that is consistent with the interpretation that these therapeutic benefits are associated with an upstream effect on the CK2α/SMO complex, moving beyond the general anti-inflammatory or antioxidant mechanisms often attributed to natural products and suggesting a new potential strategy for the targeted therapy of liver fibrosis.

The Hh pathway plays well-established roles in fibrosis, with Gli1 serving as a key transcriptional regulator of downstream genes [51,52]. Our findings illuminate a compelling mechanistic synergy: by inhibiting the upstream CK2α/SMO/Gli1 axis, miR-55 orchestrates the coordinated suppression of two major pro-fibrotic networks. It directly inhibits HSC activation by downregulating α-SMA, and modulates hypoxic responses by reducing HIF-1α. It directly inhibits HSC activation by downregulating α-SMA, and modulates hypoxic responses by reducing HIF-1α. This downregulation of HIF-1α, an established transcriptional activator of VEGF [53,54], coupled with the inhibition of the Gli1 pathway, provides a coherent mechanistic explanation for the simultaneous suppression of the pro-angiogenic factors VEGF and PDGF, effectively interrupting the pathogenic crosstalk within the HIF-1α/VEGF/PDGF axis. This inhibition of the Gli1 pathway converges with the suppression of the *TGF-β1*/*CTGF* axis, a primary driver of ECM deposition.

Beyond the Hh and angiogenic pathways, the present study provides crucial evidence that miR-55 exerts its anti-fibrotic effect by concomitantly targeting the inflammatory-fibrotic axis. The significant downregulation of TNF-α and IL-6 indicates that miR-55 intervention mitigates the chronic inflammatory milieu that serves as a primary driver for HSC activation and *TGF-β1* production. This, combined with the direct suppression of the *TGF-β1*/*CTGF* fibrogenic cascade and ECM components, demonstrates that miR-55 orchestrates a multi-faceted attack on the fibrotic process. The coordinated suppression of both upstream inflammatory triggers and downstream fibrogenic effectors is consistent with the idea that modulating the key node CK2α/SMO can disrupt a broad pathogenic network, offering a distinct advantage over single-target approaches. The VEGF/PDGF axis forms a vicious cycle in fibrosis wherein VEGF-induced angiogenesis exacerbates hypoxia, thereby inducing PDGF expression, which further stimulates HSC proliferation and VEGF secretion.

Although this work provides compelling evidence supporting the anti-fibrotic role and mechanism of miR-55, several limitations must be acknowledged. First, the MCD diet model, while effective in inducing fibrosis, causes significant weight loss, which is inconsistent with most human MAFLD phenotypes. Future studies should validate these findings in models that better recapitulate human disease pathophysiology, such as high-fat diet models or human liver organoids. Second, although we demonstrated that miR-55 promotes SMO ubiquitination and degradation, the specific E3 ubiquitin ligase involved remains unidentified. The identification of SMO-interacting E3 ligases through Co-IP coupled with mass spectrometry will be an essential next step. Furthermore, while our bioinformatic prediction and the concordant downregulation of CK2α mRNA and protein provide strong circumstantial evidence, the direct binding of miR-55 to the 3′UTR of CK2α mRNA awaits final confirmation by future experiments such as the dual-luciferase reporter assay. Finally, as Ge Xia Zhu Yu Decoction is a complex formulation, its efficacy likely arises from synergies among multiple constituents. Future research should therefore extend beyond single-component studies to systematically evaluate miR-55 in combination with other active compounds. Leveraging systems biology and AI-assisted multi-omics analyses will help elucidate network-level mechanisms and facilitate the development of optimized combination therapies with enhanced efficacy and reduced toxicity.

## 4. Materials and Methods

### 4.1. Experimental Animals and Modeling

All animal experiments were conducted in accordance with the National Institutes of Health Guide for the Care and Use of Laboratory animals and were approved by the Heilongjiang University of Chinese Medicine (Ethical Approval No. 2021052701). A total of fifty SPF SD rats were randomly assigned to five groups (*n* = 10 per group): normal control (Normal), model (MCD + Veh), miRNA negative control (MCD + NC), miR-55 intervention (MCD + miR-55), and Ge Xia ZhuYu Decoction (MCD + GXZY). The Normal group received a standard diet (Laboratory Rodent Diet 1010012, providing 3.5 kcal/g with adequate methionine and choline), while all other groups were fed a methionine-choline-deficient (MCD) diet (Dyets Inc., Bethlehem, PA, USA, Cat. No. A02082002BR; providing 3.4 kcal/g, and entirely devoid of methionine and choline) for 8 weeks to establish the MAFLD-related liver fibrosis model. Food intake was measured daily per cage, and body weight was monitored weekly. The MCD diet was provided ad libitum.

### 4.2. Oral Delivery of miR-55

The oral delivery of synthetic miR-55 mimics was achieved using a proprietary delivery system based on LNPs, developed under two national authorized invention patents [20,21]. This system ensures the stability of the miRNA throughout the gastrointestinal tract and facilitates its efficient intestinal absorption and systemic bioavailability. Briefly, miR-55 was encapsulated into these LNPs. For administration, the LNP-formulated miR-55 was prepared in a vehicle solution. Rats in the miR-55 group received a daily dose of 0.1 nmol miR-55 per rat via oral gavage in a volume of 5 mL/kg body weight. Based on the average body weight of the rats during the intervention period (approximately 250 g), this equates to a dose of approximately 0.4 nmol/kg or 2.8 μg/kg. Briefly, miR-55 was encapsulated into these LNPs and administered to rats in the miR-55 group by oral gavage at a dose of 0.1 nmol/day. The scrambled control miRNA for the MCD + NC group was delivered using the same patented system and at an equivalent volume.

### 4.3. Intervention and Monitoring

Throughout the 8-week modeling period, the following interventions were administered daily via oral gavage: the Normal and MCD + Veh received an equal volume of sterile water; the MCD + GXZY group was given an aqueous extract of Ge Xia ZhuYu Decoction (0.5 g/mL, equivalent to 0.75 g crude drug per mL) at a volume of 10 mL/kg, resulting in a daily dose of 7.5 g crude drug per kg body weight. This dosage is within the effective range (3.12–10 g crude drug/kg) established in previous preclinical studies of this formula [26,27,28]; the MCD + NC group received the control miRNA; and the MCD + miR-55 group was administered synthetic miR-55 mimics at the dose specified in Section 4.2. Body weight and general health status were monitored daily.

### 4.4. Sample Collection

At the end of the treatment period, rats were anesthetized with 1% sodium pentobarbital (Beijing Solarbio Science & Technology Co., Ltd., Beijing, China). Blood samples were collected and centrifuged at 4 °C to isolate serum, which was aliquoted and stored at −80 °C. Liver tissues were excised: one portion was fixed in 4% paraformaldehyde for subsequent histopathological evaluation, and the remaining tissue was snap-frozen in liquid nitrogen and stored at –80 °C for further molecular analyses.

### 4.5. Biochemical Analysis

Serum levels of ALT, AST, TG, TC, PCIII, IV-C, LN, HA, MDA, and total SOD activity were measured using commercially available kits (Nanjing Jiancheng Bioengineering Institute, Nanjing, China) according to the manufacturer’s instructions. Absorbance was measured using a microplate reader (SpectraMax M2, Molecular Devices, San Jose, CA, USA).

### 4.6. Liver Histopathological Analysis

Fixed liver specimens were embedded in paraffin, sectioned at 5 μm thickness, and stained with H&E, Sirius red, and Masson’s trichrome. Histopathological evaluation was performed independently by two pathologists blinded to the experimental group allocation. To quantify fibrosis, collagen deposition in Sirius red and Masson’s trichrome-stained sections was analyzed using ImageJ software (version 1.54, National Institutes of Health, Bethesda, MD, USA) by measuring the positively stained area (red for Sirius red, blue for Masson’s) relative to the total tissue area in at least five random fields per section.

### 4.7. RNA Extraction and Quantitative Real-Time PCR (qPCR)

Total RNA was isolated from liver tissues using TRIzol reagent (Thermo Fisher Scientific, Waltham, MA, USA). For the detection of mature miR-55 and U6 small RNA, reverse transcription was performed using the miRNA First Strand cDNA Synthesis (Stem-loop Method) kit (TaKaRa, Dalian, China), followed by quantitative PCR with the miRNA Quantitative PCR kit (TaKaRa), according to the manufacturer’s instructions. This stem-loop RT-qPCR method employs a universal reverse primer provided by the kit and gene-specific forward primers. For the analysis of all mRNA transcripts, reverse transcription was carried out with the Trans Script^®^ One-Step gDNA Removal and cDNA Synthesis Super Mix (Trans Gen Biotech, Beijing, China). Quantitative PCR for all targets was performed using SYBR Green Premix (Trans Gen Biotech) on a Quant Studio™ 5 Real-Time PCR System (Applied Biosystems, Foster City, CA, USA). The 2^−ΔΔCt^ method was applied to calculate relative gene expression levels. The expression of miR-55 was normalized to U6 snRNA, while mRNA levels were normalized to GAPDH. Primer sequences used are listed in Table 1.

### 4.8. Western Blot Analysis

Total protein was extracted from liver tissue using RIPA lysis buffer supplemented with 1 mM PMSF. Protein concentration was quantified using the BCA assay. Equal amounts of protein were separated by SDS-PAGE, transferred to PVDF membranes, and incubated with primary antibodies against CK2α, SMO, Gli1, and GAPDH, followed by HRP-conjugated secondary antibodies. Protein bands were visualized using an ECL chemiluminescence kit (Thermo Fisher Scientific, Waltham, MA, USA).

### 4.9. Co-Immunoprecipitation (Co-IP) and Ubiquitination Assay

Liver tissue protein lysates (300 μg) were incubated overnight at 4 °C with anti-CK2αor anti-SMO antibodies together with Protein A/G magnetic beads (Thermo Fisher Scientific). After washing, the immunoprecipitates were analyzed by Western blotting. For the ubiquitination assay, SMO was immunoprecipitated, and the membrane was probed with an anti-ubiquitin antibody.

### 4.10. Hydroxyproline Content Assay

Hepatic hydroxyproline content, as a direct measure of total collagen content, was quantified using a Hydroxyproline Assay Kit (Nanjing Jiancheng Bioengineering Institute, Nanjing, China) according to the manufacturer’s instructions. Briefly, approximately 50 mg of liver tissue was hydrolyzed, and the hydroxyproline content was determined colorimetrically and normalized to the tissue weight.

### 4.11. In Vitro Gastrointestinal Stability Assay

The stability of unmodified miR-55, 2′-F-modified miR-55, and LNP-encapsulated 2′-F-miR-55 in simulated gastrointestinal fluids was assessed as previously described in our patented delivery system with modifications [20,21]. Briefly, the three different miR-55 formulations were incubated at 37 °C in simulated gastric fluid (SGF, pH 2.0) and simulated intestinal fluid (SIF, pH 6.8). Aliquots were taken at 0, 5, 15, 30, 60, and 120 min. The reaction was terminated, and RNA was extracted. The integrity of miR-55 was assessed by stem-loop RT-qPCR, with the per-centage of intact miR-55 remaining calculated relative to the 0 min time point for each respective formulation.

### 4.12. Bioinformatics Analysis

The 3′-UTR sequence of human CK2α mRNA (CSNK2A1) was analyzed using the psRobot and RNAhybrid algorithms [55,56]. Predictions were performed under stringent criteria (psRobot score ≤ 3.0 and RNAhybrid minimum free energy ≤ −25 kcal/mol) [57].

### 4.13. Statistical Analysis

All quantitative data are expressed as mean ± standard deviation (SD). Statistical analyses were performed using GraphPad Prism software (version 9.0). Differences among multiple groups were assessed by one-way analysis of variance (ANOVA), followed by Tukey’s post hoc test. A *p*-value < 0.05 was considered statistically significant.

## 5. Conclusions

This study provides evidence that plant-derived miR-55, delivered via a gatrointestinally stable and patented oral system, exerts its potent anti-fibrotic effect through a mechanism associated with disruption of the CK2α/SMO protein–protein interaction, leading to the significant reversal of MAFLD-related liver fibrosis. The anti-fibrotic efficacy is robustly supported by a multi-dimensional assessment, including quantitative histomorphometry, a marked reduction in hepatic hydroxyproline content, and the coordinated downregulation of key fibrogenic mediators (*Col1a1*, *Col3a1*, *TIMP-1*, *TGF-β1*, and *CTGF*). Mechanistically, the therapeutic effect is consistent with a model involving a novel complex disruption-promoted degradation pathway: The suppression of CK2α expression upon miR-55 treatment is associated with disruption of the endogenous CK2α–SMO complex and promotion of its ubiquitin-mediated degradation, thereby orchestrating the coordinated suppression of the downstream Hedgehog/Gli1 signaling axis and its associated pro-fibrotic and pro-angiogenic network. These findings provide, for the first time, insights into a cross-kingdom activity of a plant miRNA that is linked to the impairment of a critical protein–protein interaction interface. Our work not only offers a mechanistic perspective on the pharmacological basis for the efficacy of Ge Xia Zhu Yu Decoction but also highlights the potential of orally delivered plant miRNAs as a promising class of therapeutics targeting diseases driven by challenging protein interactions.

## 6. Patents

The oral delivery technology and the therapeutic application of miR-55 described in this study are protected by the following Chinese national invention patents:

(1) Wang, R.; Yang, J.; Zhang, J.H.; Zhang, B.B.; Zhao, Y.W. A miR-55 Preparation, Its Preparation Method and Application. Chinese Patent ZL202211446002.4, 9 April 2024.

(2) Yang, J.; Wang, R.; Zhang, J.H.; Zhao, Y.W.; Zhang, B.B. Application of miR-55 in Inhibiting Nonalcoholic Fatty Liver Fibrosis. Chinese Patent ZL202211445995.3, 13 December 2024.

## Figures and Tables

**Table 1:** Primer Sequences.

Gene Name	Forward Sequence (5′ to 3′)	Reverse Sequence (5′ to 3′)
*MiR55*	TCGCAGGGCCGTCTTAGCTCAG	*
*U6*	CTCGCTTCGGCAGCACA	*
*GAPDH*	TGATGGGTGTGAACCACGAG	AGTGATGGCATGGACTGTGG
*CK2α*	ATGTGGTGGAATGGGGGAATC	GCAAGTGTGATGATGTTGGGC
*SMO*	ATGCGTGTTTCTTTGTGGGC	ACACAGGATAGGGTCTCGCT
*Gli1*	AGCGTGAGCCTGAATCTGTG	CAGCATGTACTGGGCTTTGAA
*HIF-1α*	GTGACCGTGCCCCTACTATG	CGTAACTGGTCAGCTGTGGT
*VEGF*	AGGGTCAAAAACGAAAGCGC	CGCGAGTCTGTGTTTTTGCA
*PDGF*	TGGAGTCGAGTCGGAAAGC	GCACTGCACATTGCGGTTA
*α-SMA*	TAGAACACGGCATCATCACC	AAGGTCGGATGCTCCTCTG
*TNF-α*	CCAGGAGAAAGTCAGCCTCCT	TCATACCAGGGCTTGAGCTCA
*IL-6*	GAGCCCACCAGGAACGAAAG	GGAAATTGGGGTAGGAAGGA
*TGF-β1*	CTCCCGTGGCTTCTAGTGC	GCCTTAGTTTGGACAGGATCTG
*TIMP-1*	CTTCTGCAATTCCGACCTCGT	ACCTGATCCGTCCACAAACAG
*Col1a1*	GAGCGGAGAGTACTGGATCG	TACTCGAACGGGAATCCATC
*Col3a1*	CTGTAACATGGAACCTGGCGA	CCATAGCTGAACTGAAAACACC
*CTGF*	GGGCCTCTTCTGCGATTTC	ATCCAGGCAAGTGCATTGGT

Note: * Universal reverse primer provided with the stem-loop RT-qPCR kit (Ta Ka Ra).

## Data Availability

The original contributions presented in this study are included in the article/Supplementary Material. Further inquiries can be directed to the corresponding author.

## Supplementary Materials

The following supporting information can be downloaded at: https://www.mdpi.com/article/10.3390/ijms27020748/s1.

## Abbreviations

MAFLD	Metabolic Dysfunction-Associated Fatty Liver Disease
MCD	Methionine-Choline-Deficient
miRNA	MicroRNA
CK2α	Casein Kinase 2 Alpha
SMO	Smoothened
Gli1	Glioma-Associated Oncogene Homolog 1
Hh	Hedgehog
α-SMA	Alpha-Smooth Muscle Actin
HIF-1α	Hypoxia-Inducible Factor 1-Alpha
VEGF	Vascular Endothelial Growth Factor
PDGF	Platelet-Derived Growth Factor
ECM	Extracellular Matrix
PPI	Protein–Protein Interaction
ALT	Alanine Aminotransferase
AST	Aspartate Aminotransferase
TG	Triglyceride
TC	Total Cholesterol
PCIII	Type III Procollagen
IV-C	Type IV Collagen
LN	Laminin
HA	Hyaluronic Acid
MDA	Malondialdehyde
SOD	Superoxide Dismutase
qPCR	Quantitative Real-Time Polymerase Chain Reaction
Co-IP	Co-Immunoprecipitation
Ub	Ubiquitin
WB	Western Blot
H&E	Hematoxylin and Eosin
SD	Standard Deviation
ANOVA	Analysis of Variance
SPF	Specific-Pathogen-Free
SD Rat	Sprague Dawley Rat
Normal	Normal diet control group
MCD + Veh	MCD diet + Vehicle
MCD + NC	MCD diet + Negative Control miRNA
MCD + miR-55	MCD diet + miR-55 mimics
MCD + GXZY	MCD diet + Ge Xia Zhu Yu Decoction
LNP	Lipid Nanoparticle
TNF-α	Tumor Necrosis Factor-Alpha
IL-6	Interleukin-6
HSC	Hepatic Stellate Cell
*Col1a1*	Collagen Type I Alpha 1 Chain
*Col3a1*	Collagen Type III Alpha 1 Chain
*TGF-β1*	Transforming Growth Factor-Beta 1
CVF	Collagen Volume Fraction
SGF	Simulated Gastric Fluid
SIF	Simulated Intestinal Fluid
IB	Immunoblot
ROS	Reactive oxygen species
