# Supplementary material for: Plant-Derived miR-55 Alleviates Liver Fibrosis by Disrupting the CK2α/SMO Complex and Promoting SMO Ubiquitination
Source: Int J Mol Sci. 2026 Jan 12;27(2):748. doi: 10.3390/ijms27020748 (PMC12840694; doi:10.3390/ijms27020748)
Supplement: Supplementary file 1 [file ijms-27-00748-s001.zip › ijms-4046373-supplementary.pdf]

Supplementary file

(A) Sequence Alignment Output from RNAhybrid

```
CSNK2A1  5' C        UG     UCUGCAUU      U               A 3'
              UACC    UG                GCU   AGAUGGC
              AUGG    AC                CGA   UCUGCCG
miR55       3'       UG     U             U                 5'
```

(B) Table of Key Prediction Parameters

Parameter	Value	Brief Interpretation
Target Gene	CK2α(CSNK2A1)	Casein Kinase 2 Alpha
Genomic Context	3′-Untranslated Region	Post-transcriptional regulatory site
Primary Prediction Tool	RNAhybrid	Algorithm for finding miRNA target sites
Minimum Free Energy	-30.4 kcal/mol	Indicates a highly stable predicted duplex. Values ≤ -25 kcal/mol are often considered strong.
Validation Tool / Score	psRobot /1.5	An independent algorithm; scores ≤ 3.0 typically denote high-confidence predictions.
Genomic Context	3′-Untranslated Region	Post-transcriptional regulatory site
Primary Prediction Tool	RNAhybrid	Algorithm for finding miRNA target sites

Figure S1. Bioinformatics prediction of miR-55 binding to the 3′-UTR of human CK2α mRNA. **(A)** Raw sequence alignment output from the RNAhybrid algorithm, depicting the predicted duplex formation between the target site within the human CK2α 3′-untranslated region (3′-UTR) (top sequence) and the plant-derived miR-55 (bottom sequence). In this representation, potential Watson-Crick (A-U, G-C) and wobble (G-U) base pairings are inferred from the **vertical alignment** of complementary nucleotides across the two sequences, rather than by explicit symbols. The alignment demonstrates a region of extended complementarity, particularly encompassing the canonical miRNA seed region (positions 2-8 from the 5‘ end of miR-55), which is crucial for target recognition and binding specificity. **(B)** Quantitative summary of the key bioinformatic parameters used to assess the confidence of this predicted interaction. The strong negative minimum free energy (MFE) indicates a thermodynamically stable and favorable hybridization event. The supporting score from a secondary, independent algorithm (psRobot) further reinforces the prediction's reliability. **Figure S1 Legend:** Comprehensive in silico evidence supporting miR-55 as a direct regulator of CK2α. The detailed sequence alignment (A) shows a region of high complementarity, and the quantitative parameters (B) meet stringent thresholds for high-confidence target prediction. This computational

analysis formed the basis for the hypothesis that miR-55 targets CK2α, guiding subsequent experimental validation.
